# Overexpression of a tomato miR171 target gene *SlGRAS24* impacts multiple agronomical traits via regulating gibberellin and auxin homeostasis

**DOI:** 10.1111/pbi.12646

**Published:** 2016-11-04

**Authors:** Wei Huang, Shiyuan Peng, Zhiqiang Xian, Dongbo Lin, Guojian Hu, Lu Yang, Maozhi Ren, Zhengguo Li

**Affiliations:** ^1^ Genetic Engineering Research Center School of Life Sciences Chongqing University Chongqing China

**Keywords:** Auxin, gibberellin, miR171, SlGRAS24, tomato (*Solanum lycopersicum*)

## Abstract

In *Arabidopsis*, the miR171‐GRAS module has been clarified as key player in meristem maintenance. However, the knowledge about its role in fruit crops like tomato (*Solanum lycopersicum*) remains scarce. We previously identified tomato *SlGRAS24* as a target gene of *Sly‐miR171*. To study the role of this probable transcription factor, we generated transgenic tomato plants underexpressing *SlGRAS24*, overexpressing *SlGRAS24*, overexpressing *Sly‐miR171* and expressing β‐glucuronidase (GUS) under the *SlGRAS24* promoter (pro*SlGRAS24*‐*GUS*). Plants overexpressing *SlGRAS24* (*SlGRAS24*‐OE) had pleiotropic phenotypes associated with multiple agronomical traits including plant height, flowering time, leaf architecture, lateral branch number, root length, fruit set and development. Many GA/auxin‐related genes were down‐regulated and altered responsiveness to exogenous IAA/NAA or GA_3_ application was observed in *SlGRAS24*‐OE seedlings. Moreover, compromised fruit set and development in *SlGRAS24*‐OE was also observed. These newly identified phenotypes for *SlGRAS24* homologs in tomato were later proved to be caused by impaired pollen sacs and fewer viable pollen grains. At anthesis, the comparative transcriptome results showed altered expression of genes involved in pollen development and hormone signalling. Taken together, our data demonstrate that *SlGRAS24* participates in a series of developmental processes through modulating gibberellin and auxin signalling, which sheds new light on the involvement of hormone crosstalk in tomato development.

## Introduction

The GRAS family of plant proteins is responsible for regulating many aspects of growth, development and responses to the biotic and abiotic environment. GRAS family members are diverse proteins that typically have five conserved motifs in the C‐terminus (Bolle, 2004). GRAS proteins usually act as transcription factors, but not all GRAS protein functions have been described. We previously identified a tomato (*Solanum lycopersicum*) GRAS transcription factor gene *SlGRAS24* as target of tomato miR171 (Huang *et al*., 2015) and aimed to discover more about its function in tomato development here.

SlGRAS24 is phylogenetically clustered into the HAIRY MERISTEM (HAM) subfamily of GRAS genes and shows the highest sequence identity with *Arabidopsis thaliana* AtSCL6 (Huang *et al*., 2015). In 2002, a GRAS transcription factor named HAM was identified as a component of a novel non‐cell‐autonomous signalling pathway maintaining shoot indeterminacy in *Petunia hybrida*,* ham* mutants displayed arrest in lateral organ and stem production (Stuurman *et al*., 2002). In the same year, two *Arabidopsis* orthologs of *Petunia HAM* were proved to be endogenous targets of post‐transcriptional degradation by *miR171*, a member of a miRNA family conserved in different plant species (Llave *et al*., 2002). Actually, a total of three *GRAS* genes are regulated by *miR171* in *Arabidopsis*,* SCL6/SCL6‐IV*,* SCL22/SCL6‐III* and *SCL27/SCL6‐II* (also known as the *HAM* or *LOM* (*LOST MERISTEMS*) genes because of their mutant phenotypes) (Reinhart *et al*., 2002). *LOM1* and *LOM2* genes promote incorporation of peripheral zone cells into leaf primordia and help to maintain a polar organization of the shoot meristem (Schulze *et al*., 2010). Further research showed that these miR171 target genes were not only required for shoot apical meristem maintenance, but for maintenance of root indeterminacy (Engstrom *et al*., 2011). More recently, it is found that HAM proteins act as conserved interacting cofactors with WUS/WOX proteins. They share common targets and their physical interaction is important in driving downstream transcriptional programmes and in promoting shoot stem cell proliferation (Zhou *et al*., 2015). *Arabidopsis* overexpressing *miR171* and the triple *scl6* mutants have similar pleiotropic phenotypes, where shoot branching, plant height, chlorophyll accumulation, primary root elongation, flower structure, and leaf shape and patterning were all altered (Wang *et al*., 2010). In barley and rice, overexpression of *miR171* affects phase transitions and floral meristem determinacy (Curaba *et al*., 2013; Fan *et al*., 2015). miR171‐GRAS module controls flowering time (phase transition) and trichome distribution via inhibiting the activity of miR156‐targeted SPL proteins (Xue *et al*., 2014). This module is also critical for mediating GA‐DELLA signalling in the coordinate regulation of chlorophyll biosynthesis and leaf growth in light (Ma *et al*., 2014). Moreover, it has been extensively studied about the role of miR171 upon various stresses in different species, including *Arabidopsis*, barley, maize and *Solanum tuberosum* (Hwang *et al*., 2011; Kantar *et al*., 2010; Kong *et al*., 2010; Liu *et al*., 2008).

HAM gene function may be conserved but the dramatic expansion in HAM homologs diversity in flowering plants strongly suggests the evolution of novel functions or functional subspecialization in angiosperms (Wu *et al*., 2014). Indeed, elevated rates of evolution in flowering plant HAM homologs indicate a refinement of HAM function in response to selective pressures (Engstrom *et al*., 2011). In tomato, six *SlGRAS* genes are clustered into the HAM subfamily, including *SlGRAS24* and *SlGRAS40*, which are confirmed to be targeted for mRNA cleavage by *miR171*, and *SlGRAS8*, a suspected target gene whose translation is repressed by *miR171* (Huang *et al*., 2015). *SlGRAS24* contains a conserved MIR‐binding sequence which is perfectly matched with *Sly‐miR171* (Huang *et al*., 2015). Despite the close evolutionary relationship between *SlGRAS24* and *SlGRAS40*, their transcripts in different tissues/organs and in response to hormone and abiotic stress differ greatly (Huang *et al*., 2015), indicating that they might have different functions and participate in distinct physiological processes.

In this study, the tomato GRAS transcription factor gene *SlGRAS24*, an ortholog of *Arabidopsis AtSCL6* (Huang *et al*., 2015), was characterized. First, the expression pattern of the gene was studied in wild‐type (WT) and transgenic plants expressing β‐glucuronidase (GUS) under the *SlGRAS24* promoter (pro*SlGRAS24*‐*GUS*). Transgenic tomato plants overexpressing *SlGRAS24* exhibited phenotypes similar to those observed in other species suggesting functional conservation among these homologs. Some new characteristics such as abnormal axillary bud emergence, reduced fruit set ratio, arrested fruit and seed development were also observed, indicating that *SlGRAS24* has additional specific functions in tomato. Moreover, the promoter of *SlGRAS24* was analysed and found to be associated with both gibberellin and auxin signalling pathways. Hormone‐related transcripts were thus quantified and the floral transcriptome was analysed in plants overexpressing *SlGRAS24*. Collectively, our study of SlGRAS24 protein unravels its role in vegetative growth and reproductive tissues and advances our understanding about hormone crosstalk in tomato.

## Results

### *Sly‐miR171* and *SlGRAS24* are ubiquitously but differentially expressed in tomato

**Figure 1:**
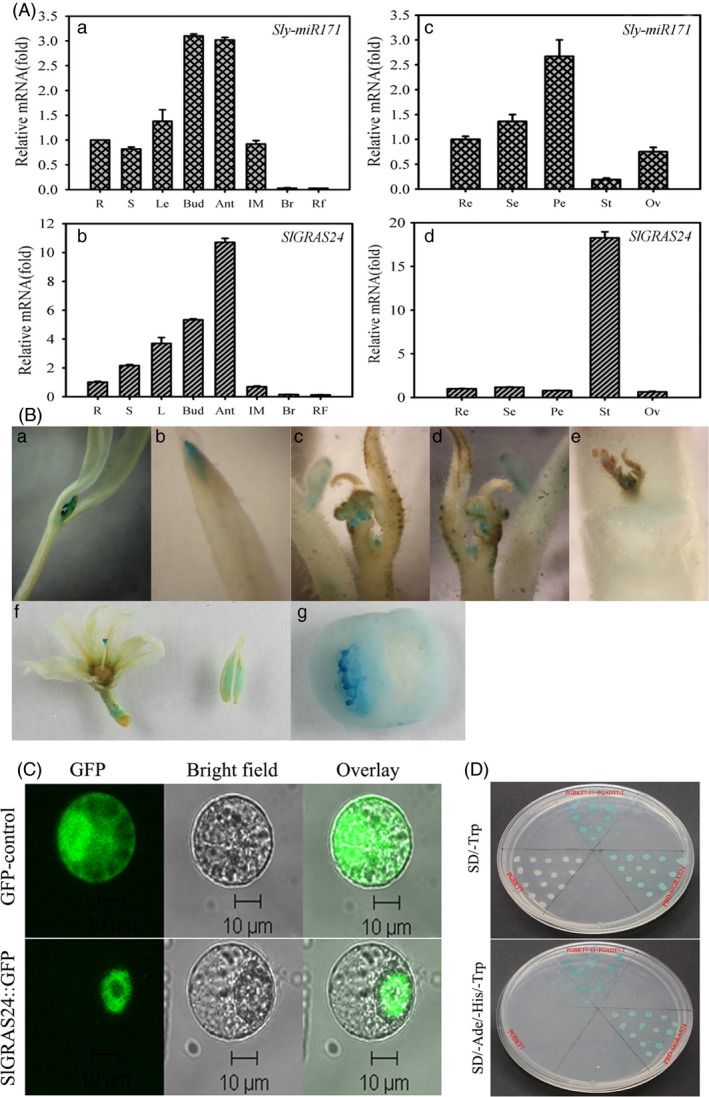
Expression patterns, subcellular localization and transcriptional activity of SlGRAS24. A, Tissue profiling analysis of *Sly‐miR171* (a, c) and *SlGRAS24* (b, d) in different organs of wild‐type tomato. R, root; S, stem; L, leaf; Bud, bud flower; Ant, anthesis flower; IM, immature green fruit; Br, colour breaker fruit; RF, ripening fruit; Re, receptacle; Se, sepal; Pe, petal; St, stamen; Ov, ovary. The expression data of root and receptacle were normalized to 1, respectively. Error bars show the standard error between three biological replicates performed (n = 3). B, Expression patterns of *SlGRAS24* via GUS staining: (a, b) young seedlings; (c, d) shoot apices of phase transition stage plants; (e) nodal stem and axillary buds; (f) anthesis flowers; (g) immature green fruits. C, Subcellular localization of SlGRAS24. The photographs were taken under bright light, in the dark field for the GFP‐derived green fluorescence and merged, respectively. D, Analysis of the transactivation activity of SlGRAS24. Up, SD/‐Trp medium; below, SD/‐Ade/‐His/‐Trp medium; both contained X‐α‐gal for assaying another yeast reporter (*MEL1*) gene.

We hypothesized that the *SlGRAS24* gene and its regulator *Sly‐miR171* would have similar functions in tomato as their respective orthologs in *Arabidopsis* (Wang *et al*., 2010). We investigated this first by quantifying the expression of *Sly‐miR171* and *SlGRAS24* in the ‘Micro‐Tom’ cultivar (WT) by qRT‐PCR. Both genes were detectable in all tissues with the highest expression levels found in flowers (Fig. 1A). The levels of both transcripts dropped during fruit development and the lowest level of expression was at the ripening stage. Overall, *Sly‐miR171* and *SlGRAS24* mRNAs had similar transcription patterns, which were consistent with research on their *Arabidopsis* orthologs (Wang *et al*., 2010). However, floral organs were an exception because *SlGRAS24* mRNA was most abundant in stamens where *Sly‐miR171* mRNA expression was at its lowest level. The expression data here suggest that miR171‐SlGRAS24 regulatory networks are needed throughout vegetative and reproductive development in tomato.

Spatial expression of *SlGRAS24* was monitored by histochemical staining of transgenic tomato in which GUS reporter's expression was driven by the upstream promoter sequence of *SlGRAS24* gene (Fig. 1B). In pro*GRAS24*‐*GUS* homozygous seedlings, GUS reporter activity was strong in leaf primordia and root tips, but quite weak in cotyledons and hypocotyls. GUS activity was also expressed in flower primordia, in the young leaves’ margins of shoot apices, in internodes and in axillary buds. In reproductive tissues, *SlGRAS24* was highly expressed in stamens and stigmas, and predominantly expressed in seeds of young fruits. These results suggest that *SlGRAS24* expression is spatiotemporally regulated and *SlGRAS24* may have specific functions in developmental processes.

### SlGRAS24 is a transcription factor targeted to the nucleus

To determine the subcellular localization of SlGRAS24 protein, the vector 35S‐*SlGRAS24*‐*GFP* was transiently expressed in tobacco protoplasts. Confocal imaging of protein fluorescence showed that the green fluorescence signal of 35S‐*SlGRAS24*‐*GFP* was exclusively detected in the nucleus, whereas the cells transformed with the vector containing *GFP* alone displayed fluorescence throughout the cells (Fig. 1C). A yeast two‐hybrid experiment was used to examine the transcriptional activity of SlGRAS24. A GAL4 DNA‐binding domain SlGRAS24 fusion protein was expressed in yeast cells, which were then assayed for their ability to activate transcription from the GAL4 sequence. SlGRAS24 promoted yeast growth in the absence of histidine and adenine, and showed X‐α‐gal activity, whereas the control vector pGBKT7 did not (Fig. 1D). These results suggest that SlGRAS24 has transcriptional activity and is targeted to the nucleus in plant cells.

### Phenotypic characterization of *SlGRAS24*‐OE lines

**Figure 2:**
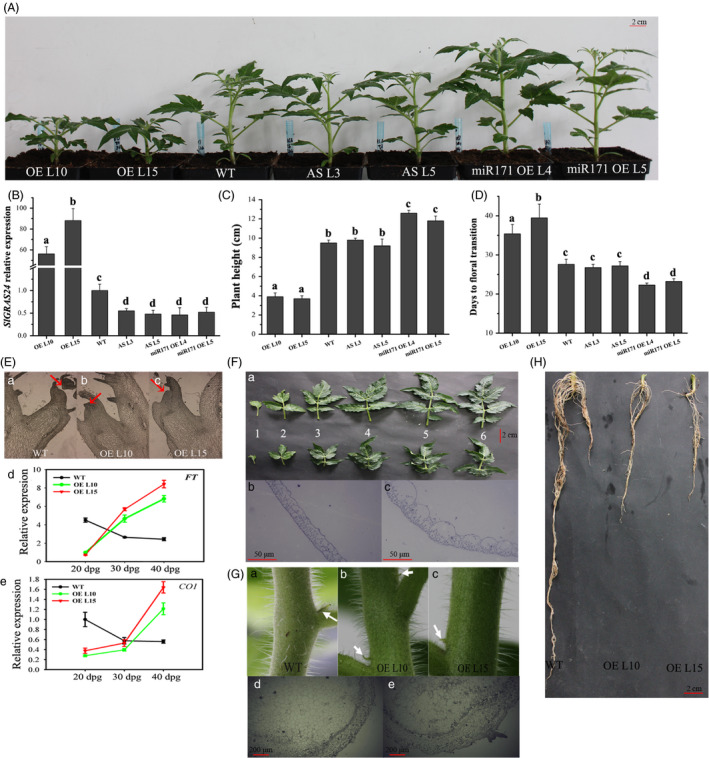
Phenotypic characterization of wild‐type and transgenic plants. A, Image of 30‐day‐old plants of different genotypes. OE L10 and OE L15, two independent *SlGRAS24*‐overexpressing lines; AS L3 and AS L5, two independent *SlGRAS24*‐downexpressing lines; miR171 OE L4 and miR171 OE L15, two independent Sly‐miR171‐overexpressing lines. B, Expression levels of *SlGRAS24* in plants shown in A. Expression of *SlGRAS24* in WT was normalized to 1. Error bars show the standard error between three biological replicates performed (n = 3). C, Height of plants shown in A. D, Days to floral transition (phase transition time) of plants shown in A. For C and D, error bars show the standard error between three biological replicates (n = 3) with more than ten plants for each replicate performed. E, Longitudinal sections of shoot apices from 25‐day‐old WT (a) and *SlGRAS24*‐OE (b, c) plants; arrows indicate the emerged flower bud (a) and flower primordium (b, c); expression analysis of two flowering time regulator *FT* (d) and *CO1* (e) in leaves from WT and *SlGRAS24*‐OE plants; dpg, days postgermination. F, Leaves from different positions (at nodes 1‐6 from cotyledons) in WT and *SlGRAS24*‐OE plants (a), transverse sections of leaves from node 5 in the WT (b) and *SlGRAS24*‐OE (c) plants. G, Axillary buds of WT (a) and *SlGRAS24*‐OE (b, c) plants; arrows indicate the positions of new formed axillary buds; transverse sections of stems in WT (d) and *SlGRAS24*‐OE (e) plants. H, Roots in WT and *SlGRAS24*‐OE plants. Different letters above bars indicate significant differences among different genotypes (Student's *t*‐test, *P* < 0.05).

To assess the physiological importance of the *SlGRAS24*‐encoded protein, the tomato ‘Micro‐Tom’ genotype was transformed with sense or antisense constructs of the *SlGRAS24* gene to produce several independent overexpressing (*SlGRAS24*‐OE) or underexpressing (*SlGRAS24*‐AS) homozygous lines. qRT‐PCR was performed to evaluate the expression of *SlGRAS24* in transgenic plants, and the results showed that the *SlGRAS24* was successfully up‐regulated or down‐regulated in all transgenic lines tested (Table 1). Thereby, two most up‐ or down‐regulated lines of each genotype were then chosen for the following characterization. Interestingly, *SlGAR24*‐AS lines did not differ from WT tomato (Fig. 2A and B). This was not unexpected as no phenotype had been observed in *Arabidopsis* plants in which only one of the *HAM* genes was mutated (Wang *et al*., 2010). We hypothesized that there is functional redundancy among GRAS family members or multiple *miR171* target genes in tomato. We tested this by generating transgenic plants overexpressing the precursor of tomato *miR171* to silence *miR171* target genes including *SlGRAS24* (Table 1). *SlGRAS24* expression was inhibited to a similar degree in *SlGAR24*‐AS and *SlymiR171*‐OE plants comparing to WT, but only *SlymiR171*‐OE demonstrated taller plants with earlier phase transition time (Fig. 2C and D). The *SlGRAS24*‐OE lines showed pleiotropic phenotypes, some of which were in line with the phenotypes of transgenic *Arabidopsis* plants overexpressing *SlGRAS24* orthologs *AtHAMs* (Wang *et al*., 2010). Flower opening was significantly delayed in *SlGRAS24‐*OE, which was consistent with the delayed phase transition time from vegetative to reproductive development. The tissue sections of apical meristems showed no formed floral primordium in *SlGRAS24‐*OE lines, 25 days postgermination (dpg) (Fig. 2E). In line with that, transcript levels of *SlFT* and *SlCO1*, two key regulators controlling flowering time, undergone absolutely opposite expression tendency in samples harvested at three different stages (20 dpg, 30 dpg, and 40 dpg) (Fig. 2E). The leaves of transgenic plants were both shorter and narrower than WT leaves and leaf margins were not serrated (Fig. 2F). Microscopic analysis showed that leaves were thicker in *SlGRAS24*‐OE lines, which might be attributed to having much larger lower epidermal cells (Fig. 2F). WT plants had axillary buds at the internodes, but abnormal axillary bud emergence was observed on stems of *SlGRAS24*‐OE plants (Fig. 2G). Besides, *SlGRAS24*‐OE plants had more lateral branches and abnormal flower bud emergence (Fig. S1). Primary and lateral root growth of *SlGRAS24*‐OE lines was strongly suppressed compared to WT (Fig. 2H). More detailed information about the phenotypes of *SlGRAS24*‐OE transgenic tomato plants is shown in Table 2. Noticeably, the fruit set ratio of *SlGRAS24*‐OE tomato plants severely decreased, and fruit development was defective (Table 2).

**Table 1:** Summary of phenotypes in transgenic tomato plants overexpressing *SlGRAS24*, underexpressing *SlGRAS24* and overexpressing *Sly‐miR171*

Genotype	Line number	*SlGRAS24* expression (relative to WT)	Phenotype
35S‐*SlGRAS24*	L1–L16	8.4–88.8 folds	Dwarf plants, delayed phase transition time, abnormal leaves, inhibited root growth, increased lateral shoots, decreased flower number, impaired fruit set, compromised fruit and seed development
35S‐as*SlGRAS24*	L1–L7	0.48–0.65 folds	No apparent phenotype
35S‐*SlymiR71*	L1–L5	0.46–0.74 folds	Higher plants, earlier phase transition time and flowering time

**Table 2:** Phenotypes of wild‐type (WT) and *SlGRAS24*‐overexpressing (*SlGRAS24*‐OE) transgenic tomato plants (L10 and L15)

Parameter	WT	OE L10	OE L15
Plant height (one month old, cm)	9.5 ± 0.3	3.9 ± 0.4**	3.7 ± 0.3**
Plant height (two months old, cm)	19.4 ± 1.1	11.1 ± 0.5**	9.6 ± 1.2**
Plant height (three months old, cm)	20.3 ± 1.9	13.8 ± 1.2**	13.1 ± 1.4**
Plant height (four months old, cm)	20.8 ± 2.5	18.1 ± 2.3	21.4 ± 2.7
Stem diameter of sixth internode (two months old, cm)	5.4 ± 0.5	5.3 ± 0.3	5.3 ± 0.3
Leave length/width of sixth node (two months old, cm)	5.8 ± 0.5/2.5 ± 0.2	4.3 ± 0.6/1.6 ± 0.4*	3.7 ± 0.5/1.3 ± 0.3**
Primary root length (two months old, cm)	36.2 ± 3.3	15.4 ± 2.8**	12.6 ± 1.9**
Lateral root number (two months old, n)	47.6 ± 4.1	26.6 ± 3.3**	14.8 ± 3.5**
Lateral branch number (two months old, n)	0.5 ± 0.2	2.2 ± 0.3**	2.9 ± 0.4**
Leaves to first inflorescence (n)	6.8 ± 0.3	6.5 ± 0.4	6.6 ± 0.2
Days to first visible flower bud	27.6 ± 1.3	35.4 ± 2.4**	39.5 ± 3.5**
Days to anthesis of first flower	42.1 ± 0.7	52.7 ± 2.6**	57.3 ± 2.8**
Days to colour breaker of first mature fruit	80.3 ± 1.9	90.9 ± 2.7**	97.2 ± 3.6**
Flowers in the two first inflorescences (n)	21.7 ± 2.1	16.0 ± 1.6**	13.2 ± 1.3**
Fruit set ratio	90 ± 1.4%	22 ± 2.3%**	15 ± 1.8%**
Fruits per plant (n)	26.3 ± 2.8	11.2 ± 2.1**	7.5 ± 1.4**
Fruit production (g per plant)	85.8 ± 10.3	24.6 ± 4.7**	11.2 ± 2.8**

Values are means of 10–12 plants, ±SE. The statistical significance of mean differences was analysed using a *t*‐test: *P < 0.05, **P < 0.01.

### Overexpression of *SlGRAS24* disrupts fertilization

**Figure 3:**
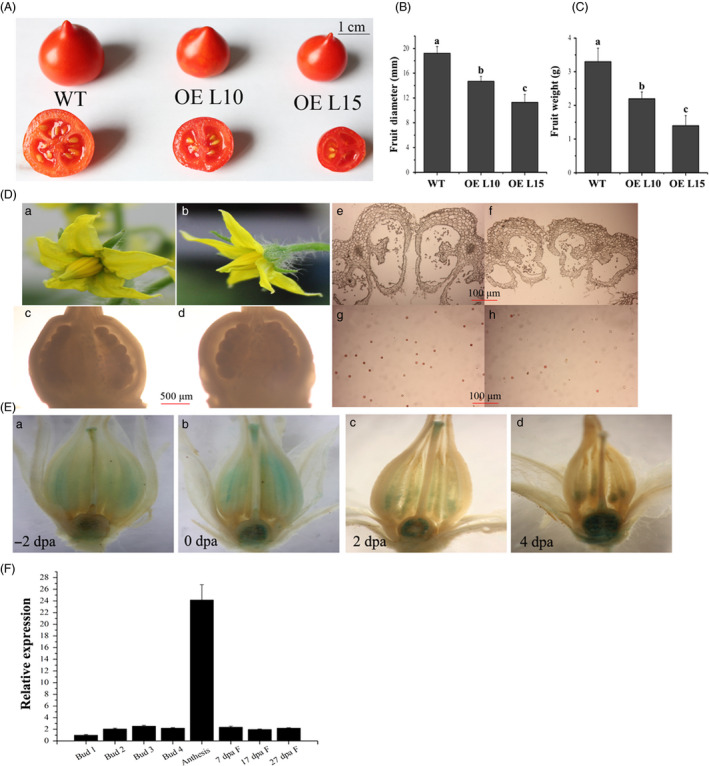
Overexpression of *SlGRAS24* causes smaller fruits with less seeds due to impaired fertilization. A, Fruits of WT and *SlGRAS24*‐OE plants. B and C, Diameter and weight of WT and *SlGRAS24*‐OE fruits. Error bars show the standard error between three biological replicates (n = 3) with more than ten fruits for each replicate performed. Different letters above bars indicate significant differences among different genotypes (Student's *t*‐test, *P* < 0.05). D, Anthesis flowers in WT (a) and *SlGRAS24*‐OE showing dehiscent stamen (b). Ovary and ovule of WT (c) and *SlGRAS24*‐OE (d) anthesis flowers. Transverse sections of anthers of WT (e) and *SlGRAS24*‐OE (f) anthesis flowers. Comparison of the pollen energy of WT (g) and *SlGRAS24*‐OE (h) with TTC staining. E, GUS staining analysis of ‐2 dpa (a), 0 dpa (b), 2 dpa (c) and 4 dpa flowers from pro*SlGRAS24*‐*GUS* transgenic plants. Dpa, days postanthesis. F, Expression level of *SlGRAS24* in flowers and fruits at different developmental stages. Buds were divided into 4 developmental stages from 1 to 4 according to the length of bud. Bud 1 stands for no more than 1 mm, bud 2 stands for between 2 and 3 mm, bud 3 stands for between 4 and 5 mm, and bud 4 stands for between 6 and 7 mm. Error bars show the standard error between three biological replicates performed (n = 3).

*SlGRAS24*‐OE plants showed reduced fruit set and fruits were smaller with fewer seeds (Table 2, Fig. 3A, B and C). As tomato is the most important model for fruit development, it is meaningful to investigate the molecular mechanism underlying these defects. We found that *SlGRAS24*‐OE plants occasionally produced flowers with dehiscent stamens, but the ovary and ovules in *SlGRAS24*‐OE flowers were as in WT (Fig. 3D). However, *SlGRAS24*‐OE flowers had smaller pollen sacs and collapsed anthers (Fig. 3D). TTC staining for pollen viability showed that fewer pollen viable grains in *SlGRAS24*‐OE flowers undergoing anthesis (first day of flower opening) than that in WT flowers (Fig. 3D). Considering that *SlGRAS24* transcripts were most abundant in anthesis flowers (Fig. 1A and B), expression pattern was studied in more detailed by examining GUS staining in flowers from ‐2 days postanthesis (dpa) to 4 dpa (Fig. 3E) and by qPCR analysing in flowers and fruits at different stages (Fig. 3F). For GUS staining, in stamens and stigmas, the strongest staining was at 0 dpa, and it became much weaker at 2 dpa, till there was almost no expression at 4 dpa. By contrast, almost no staining was observed in −2 dpa ovaries, but the expression increased since 0 dpa ovaries but was limited to ovules (Fig. 3E). Consistently, qPCR results also showed most abundant *SlGRAS24* transcript in flowers at the anthesis stage (Fig. 3F).

Cross‐fertilization assay was carried out to examine the fertility of transgenic flowers (Table 3). Fruit set ratios were 90%, 22% and 15% in self‐pollinated WT flowers, OE L10 flowers and OE L15 flowers, respectively. When WT flowers were used as the female recipient, the fruit set ratio increased slightly to 33% and 25% with OE L10 and OE L15 pollen, respectively. There was 100% fruit set when WT pollen was used to pollinate OE pistils. The results also showed that WT × WT fruits and WT × OE fruit (pollen × pistil crosses) contained more seeds than OE × WT and OE × OE fruit. These experiments demonstrate that *SlGRAS24* is necessary for normal stamen development and overexpression of *SlGRAS24* has a negative impact on fertilization in tomato.

**Table 3:** Cross‐fertilization assay. Emasculated wild‐type flowers were fertilized with *SlGRAS24*‐OE pollen and the number of fruit and number of seed in each fruit were assessed at the ripe stage. Conversely, tomato pollen from wild‐type flowers was used to fertilize emasculated *SlGRAS24*‐OE flowers. Spontaneous self‐pollinated flowers from each genotype were used as control. For each cross‐fertilization assay, the capacity of the T1 seeds to grow on kanamycin‐containing medium was assessed. Results are representative of data from two independent lines (OE L10 and OE L15)

Cross	Fruit set (Fruits Developed/No. of Attempts)	Fruit set ratio (%)	Seeds (No. per fruit)	F1 kanamycin resistance (%)
♀ WT × ♂ WT	18/20	90	22.8 ± 3.5	0
♀ OE L10 × ♂ OE L10	4/18	22	6.6 ± 2.3	100
♀ OE L15 × ♂ OE L15	3/20	15	5.5 ± 1.8	100
♀ WT × ♂ OE L10	5/14	36	5.4 ± 2.4	100
♀ WT × ♂ OE L15	4/16	25	4.6 ± 0.8	100
♀ OE L10 × ♂ WT	12/12	100	14.2 ± 2.1	100
♀ OE L15 × ♂ WT	10/10	100	11.3 ± 1.6	100

### Overexpression of *SlGRAS24* inhibits cell division and expansion in fruit

**Figure 4:**
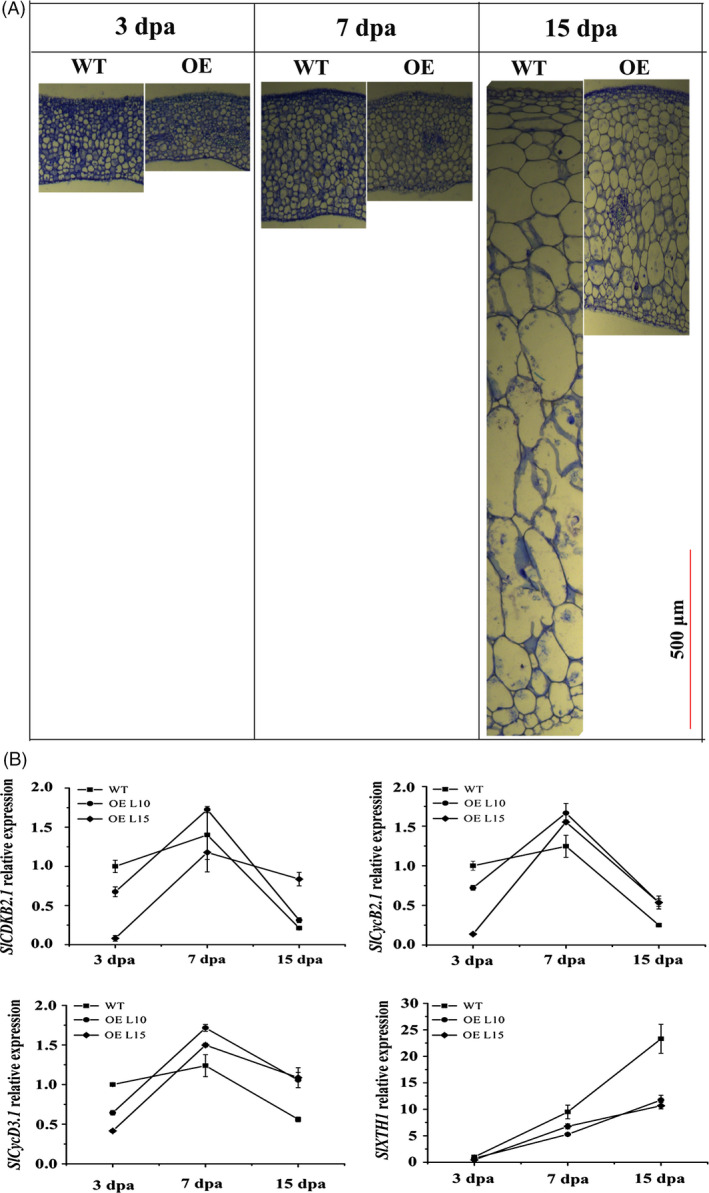
Histology and qPCR analysis of *SlGRAS24*‐OE fruits. A, Transverse sections of 3 dpa, 7 dpa and 15 dpa WT and *SlGRAS24*‐OE fruit pericarps. B, qRT‐PCR analysis of cell division and expansion genes in 3 dpa, 7 dpa and 15 dpa WT and *SlGRAS24*‐OE fruits. Dpa, days postanthesis. Error bars show the standard error between three biological replicates performed (n = 3).

As *SlGRAS24*‐OE plants have smaller fruits, the histology of WT and *SlGRAS24*‐OE ovaries was analysed from day 3 to day 15 (Fig. 4A and Fig. S2). The pericarp of *SlGRAS24*‐OE ovaries contains smaller cells and fewer cell layers than WT pericarp, indicating that overexpression of *SlGRAS24* led to an inhibition of cell division and expansion in early fruit development. We analysed transcript levels of four genes involved in cell division (*CYCLIN DEPENDENT KINASE* (*SlCDKB2.1*), *CYCLIN* (*SlCycB2.1*) and *SlCycD3.1*, which encodes a G1 cyclin) and cell expansion (*XYLOGLUCAN ENDOTRANSGLUCOSYLASE*/*HYDROLASE 1SlXTH1*) (Fig. 4B). Relative to WT ovaries, the expressions of the three cell division genes were low in 3 dpa ovaries and increased at 7 dpa and 15 dpa. By contrast, expression of the *SlXTH1* cell expansion gene was always lower in *SlGRAS24*‐OE ovaries than in WT ovaries.

### *SlGRAS24* is involved in GA and auxin signalling

**Figure 5:**
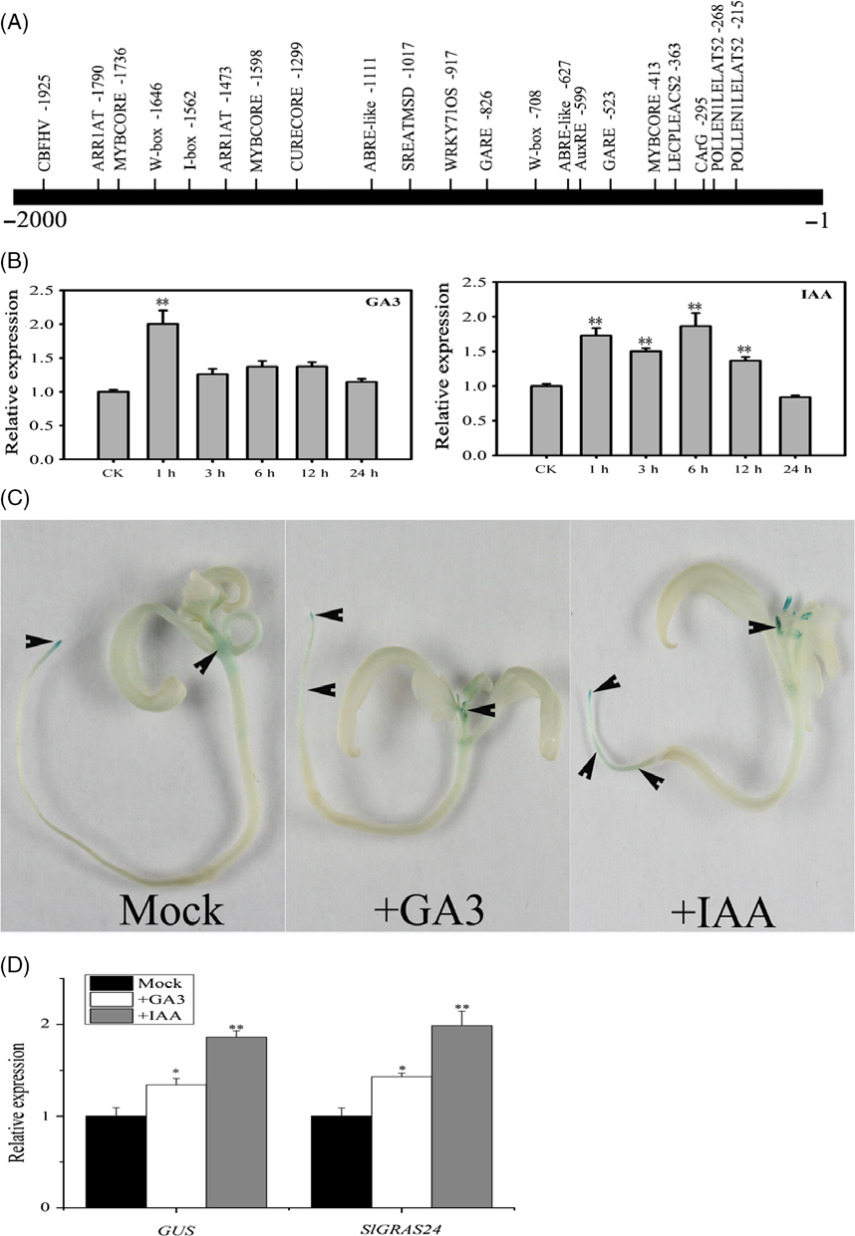
*SlGRAS24* is involved in GA and auxin signalling. A, Promoter region and the putative *cis*‐acting elements. B, qRT‐PCR analysis of *SlGRAS24* mRNA using leaves of 2‐month‐old WT plants after treatment with 100 μM GA_3_ or 100 μm IAA. Expression of *SlGRAS24* in 0‐h treated plants was normalized to 1. Different letters above bars indicate significant differences among different treatment time points (Student's *t*‐test, *P* < 0.05). C, Expression pattern of pro*SlGRAS24*‐*GUS* in 15‐day‐old seedlings and exogenous GA_3_ or IAA treatment (20 μm for 3 h); arrows indicate places with clearly GUS staining. D, qRT‐PCR analysis of *GUS* and *SlGRAS24* mRNA in 15‐day‐old pro*SlGRAS24*‐*GUS* transgenic seedlings shown in C. Expression of *SlGRAS24* or *GUS* in untreated seedlings was normalized to 1. All samples were collected at the indicted time points for three biological replicates (n = 3). Different letters above bars indicate significant differences among different genotypes (Student's *t*‐test, *P *< 0.05).

The 2.2‐kb *SlGRAS24* promoter sequence was analysed *in silico* using the PLACE program (http://www.dna.affrc.go.jp/PLACE/signalup.html). Several *cis*‐acting elements were identified including the canonical auxin response element (AuxRE) at position ‐599, two GA‐responsive elements (GARE) at positions −523 and −826, and other elements related to GA and auxin (Fig. 5A). This strongly suggests that *SlGRAS24* is regulated by both two hormones. *SlGRAS24* transcript levels were compared in leaves treated or untreated by GA_3_ or IAA, respectively. *SlGRAS24* expression increased significantly within 1 h in response to either GA_3_ or IAA (Fig. 5B). GA and auxin responsiveness of the promoters were tested using pro*GRAS24*‐*GUS* transgenic seedlings incubated in solutions containing GA_3_ or IAA for 3 h. Compared with untreated seedlings (mock), GUS staining revealed that GA_3_ or IAA treatment led to ectopic expression of the *GUS* gene (Fig. 5C). qPCR analysis showed that both *GUS* and *SlGRAS24* transcripts significantly increased in response to GA_3_ or IAA treatment in pro*GRAS24*‐*GUS* seedlings (Fig. 5D).

### Expression of GA‐ and auxin‐related genes is differently regulated in *SlGRAS24*‐OE seedlings

**Figure 6:**
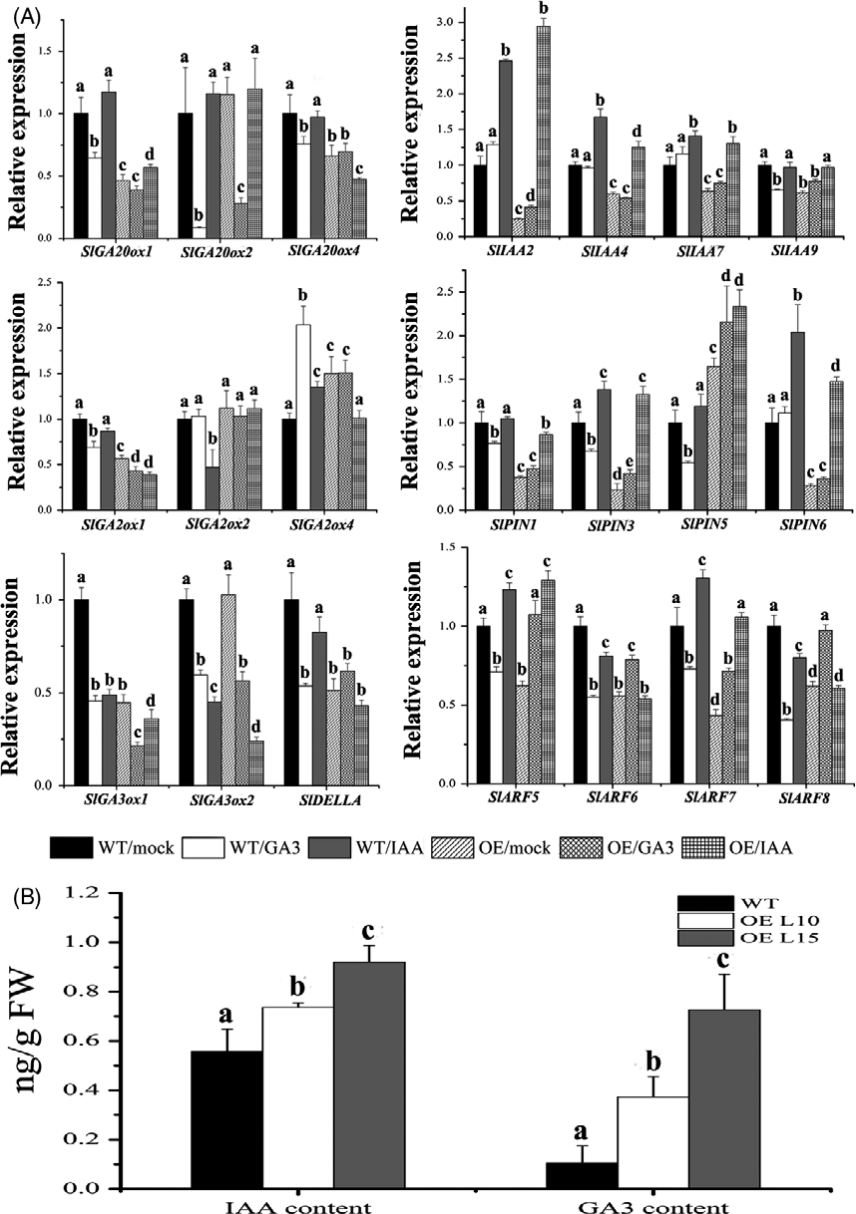
Expression analysis of GA/auxin‐related genes and endogenous IAA/GA_3_ content characterization. A, qRT‐PCR analysis of GA‐ and auxin‐related genes in 10‐day‐old WT and *SlGRAS24*‐OE seedlings as well as in response to GA_3_ or IAA treatment (20 μM for 3 h). B, Endogenous IAA and GA_3_ content in 10‐day‐old WT and *SlGRAS24*‐OE seedlings. Error bars show the standard error between three biological replicates performed (n = 3). Different letters above bars indicate significant differences among different treatments/genotypes (Student's *t*‐test, *P* < 0.05).

To further study the role of *SlGRAS24* in GA and auxin pathways, the expression levels of a panel of 21 tomato genes were monitored in WT and *SlGRAS24*‐OE seedlings in response to GA_3_ or IAA treatment (Fig. 6A). The panel was made up of 5 GA biosynthetic enzymes (*SlGA20ox1*,* SlGA20ox2*,* SlGA20ox4*,* SlGA3ox1* and *SlGA3ox2*), 3 GA deactivating enzymes (*SlGA2ox1*,* SlGA2ox2* and *SlGA2ox4*), a key regulator of GA signalling pathway (*SlDELLA*), 4 auxin/indole‐3‐acetic acid (Aux/IAA) transcription factors (*SlIAA2*,* SlIAA4*,* SlIAA4* and *SlIAA9*), 4 auxin response gene (ARF) transcription factors (*SlARF5*,* SlARF6*,* SlARF7* and *SlARF8*) and 4 PIN‐FORMED (PIN) auxin efflux transport proteins (*SlPIN1*,* SlPIN3*,* SlPIN5* and *SlPIN6*). Without hormone treatment, 17 genes were down‐regulated and 2 genes were up‐regulated in *SlGRAS24*‐OE seedlings compared to WT, which suggested that overexpression of *SlGRAS24* disrupts GA and auxin homeostasis in transgenic plants. Furthermore, some of these genes responded differently to GA_3_ and/or IAA in *SlGRAS24*‐OE seedlings as compared to WT. For instance, upon GA_3_ treatment, *SlDELLA* expression decreased in WT, but significantly increased in *SlGRAS24*‐OE seedlings. *SlGA2ox4* was induced by both GA_3_ and IAA treatment in WT, while in *SlGRAS24*‐OE it was not induced in response to GA_3_ and was inhibited in response to IAA. *SlIAA2* was up‐regulated 2.45‐fold by IAA treatment in WT, but about 12‐fold in OE seedlings. *SlARF8* was down‐regulated under both hormone treatments in WT, while in *SlGRAS24*‐OE it increased in response to GA_3_ treatment and did not respond to IAA treatment. Comparing WT and *SlGRAS24*‐OE seedlings, the different responsiveness of the GA‐related genes during IAA treatment, and conversely the auxin‐related genes during GA_3_ treatment, might indicate that *SlGRAS24* acts as an integrator between GA and auxin pathways.

It is thus possible that *SlGRAS24* plays a role in regulating the expression of hormone‐related genes in tomato, particularly genes associated with GA or auxin biosynthesis, transport and signal transduction. To assess whether changes in hormone levels accompanied changes in gene expression, endogenous IAA and GA_3_ were quantified using HPLC‐MS/MS (Fig. 6B). *SlGRAS24*‐OE seedlings contained more IAA and GA_3_ than in WT seedlings. This was somewhat unexpected as most GA/auxin‐related genes were down‐regulated.

### Altered responsiveness to GA_3_ and IAA application for *SlGRAS24*‐OE plants

**Figure 7:**
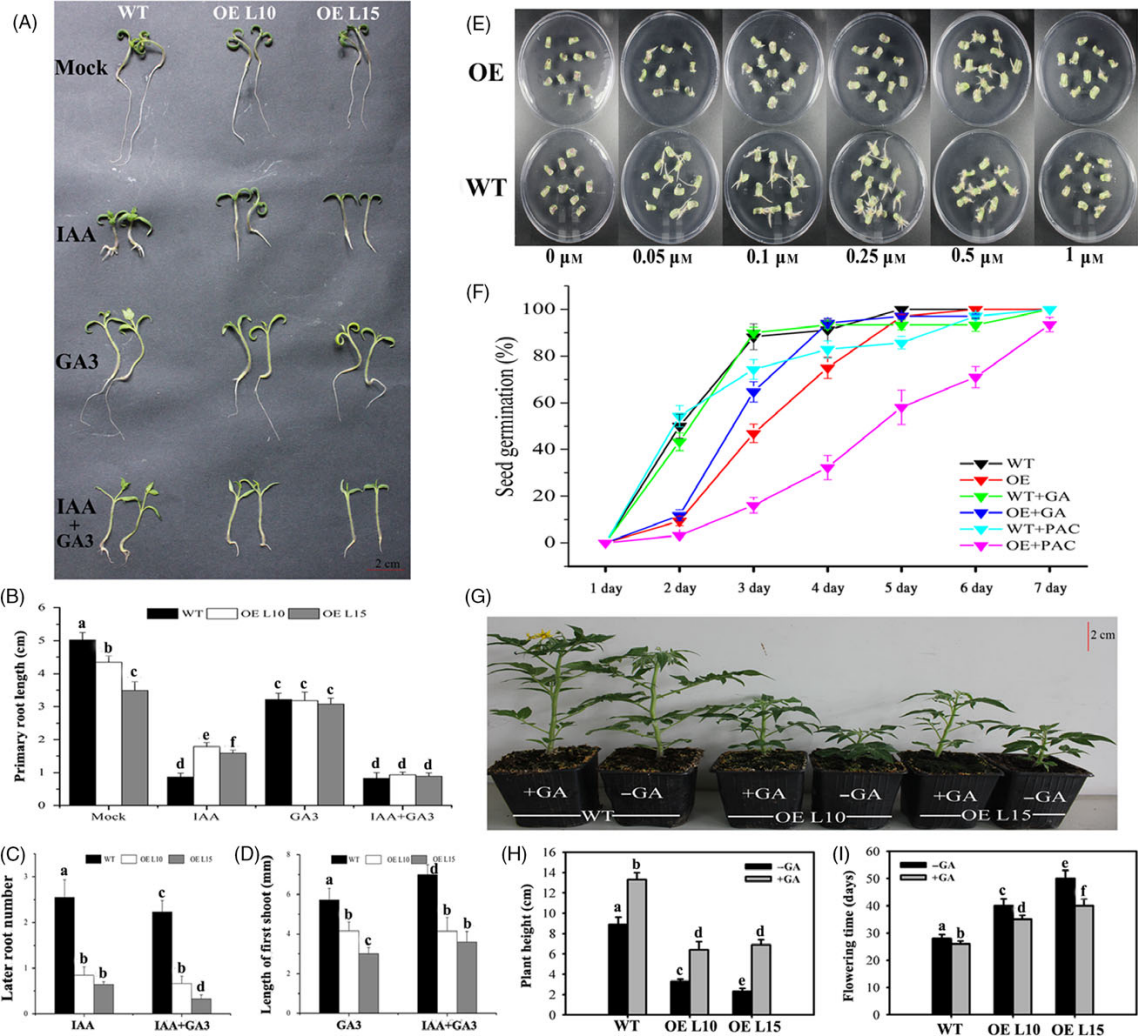
Altered responsiveness to exogenous IAA/GA_3_ for *SlGRAS24*‐OE lines. A, Phenotypes of two‐week‐old WT and *SlGRAS24*‐OE seedlings grown on MS/2 medium containing 1 μm IAA and/or 50 μm GA_3_. B, The length of primary root in WT and *SlGRAS24*‐OE seedlings shown in A. C, The number of lateral roots in WT and *SlGRAS24*‐OE seedlings under IAA or IAA+GA_3_ treatment. D, Length from hypocotyls to true leaves in WT and *SlGRAS24*‐OE seedlings under GA_3_ or IAA+GA_3_ treatment. E, Auxin dose–response assay of cotyledon explants. The explants were treated with increasing concentrations (0, 0.05, 0.1, 0.25, 0.5 and 1.0 μm) of NAA. F, Germination assay of WT and *SlGRAS24*‐OE line as well as in response to GA_3_ (10 μm) or PAC (5 μm) treatment. G, *SlGRAS24*‐OE dwarfism partially rescued by exogenous GA_3_ application. H and I, Plant height and phase transition time in GA_3_ treated plants shown in G. Error bars show the standard error between three biological replicates (n = 3) with more than ten plants for each replicate performed. Different letters above bars indicate significant differences among different genotypes (Student's *t*‐test, *P* < 0.05).

Exogenous IAA and/or GA3 were applied to WT and *SlGRAS24*‐OE seedlings to investigate whether overexpression of *SlGRAS24* altered other aspects of GA and auxin responsiveness (Fig. 7A). Without hormone treatment, the primary roots of *SlGRAS24*‐OE seedlings were distinctly shorter than those of WT. In the presence of 1.0 μm IAA treatment, primary root growth was inhibited in both WT and *SlGRAS24*‐OE seedlings (Fig. 7A and B), while *SlGRAS24*‐OE seedlings had longer primary roots and fewer lateral roots than WT seedlings (Fig. 7A, B, and C), indicating that auxin responsiveness was reduced when *SlGRAS24* was overexpressed. Synthetic auxin NAA stimulated more and longer adventitious roots to form from excised WT cotyledons than from *SlGRAS24*‐OE cotyledons (Fig. 7E), another indication of a reduced auxin response in *SlGRAS24*‐OE seedlings. Similarly, 50 μm GA3 inhibited the primary root growth of both WT and *SlGRAS24*‐OE tomato seedlings, reducing the initial difference in length (Fig. 7A and B). Besides, outgrowth of the first true leaves from the shoot apex was severely suppressed in *SlGRAS24*‐OE seedlings compared with WT (Fig. 7A and D).

Under normal condition, germination was inhibited in seeds from *SlGRAS24*‐OE compared to that from WT (Fig. 7F). More sensitive phenotypes were observed in *SlGRAS24*‐OE seeds when 10 μm GA3 or 5 μm paclobutrazol, a GA biosynthesis inhibitor, was applied since no observation of marked difference when they were applied to WT seeds (Fig. 7F), implying that *SlGRAS24* is likely involved in seed germination through modulating GA signalling. However, the germination rates were not fully recovered to WT level when GA_3_ was applied (Fig. 7F). Similarly, the dwarf phenotype and delayed flowering time of *SlGRAS24*‐OE plants were only partially rescued by spraying with 20 μm GA_3_ (Fig. 7G, H and I), suggesting that some GA‐independent pathways are involved in *SlGRAS24*‐mediated regulation of plant growth and seed germination.

### Overexpression of *SlGRAS24* causes transcriptome changes in flowers at anthesis

**Figure 8:**
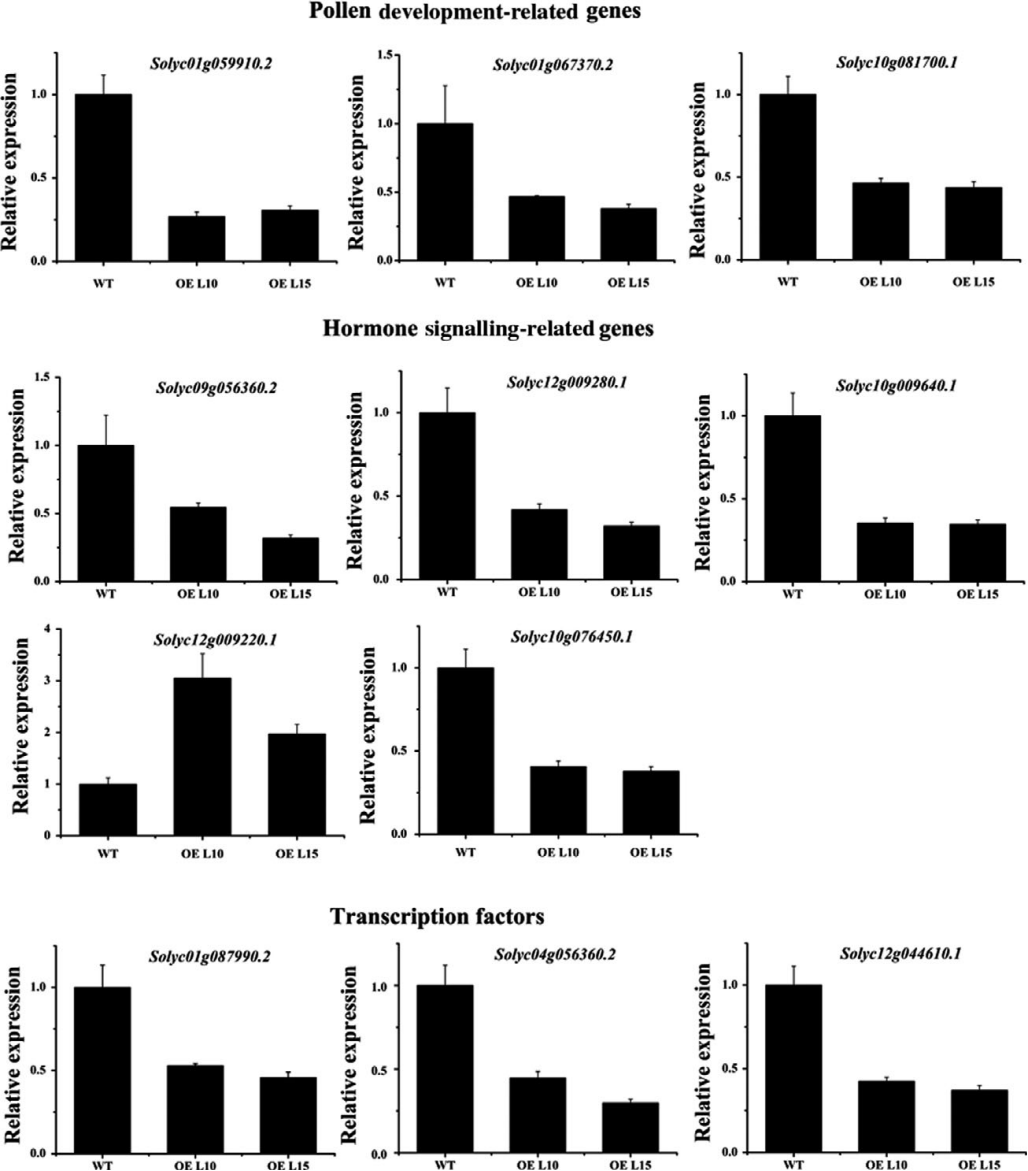
qPCR validation of transcriptomic data. 3 pollen development‐related genes, 5 hormone signalling‐related genes and 3 transcription factor genes were selected and validated by qRT‐PCR. Error bars show the standard error between three biological replicates performed (n = 3).

To detect the changes in transcript levels that may be involved in the flower–fruit transition, a comparative transcriptome analysis was conducted using flowers at the onset of anthesis (0 dpa) of two *SlGRAS24*‐OE transgenic lines (L10 and L15) and WT controls. Under the criteria of false discovery rate <0.05 and log2 fold change ≥1, a total of 1671 and 1436 unigenes were differentially expressed in L10 and L15, respectively, compared with WT controls (File S1 and S2). Functional annotation of putative gene products indicated that overexpression of *SlGRAS24* affected multiple processes including transcription, signal transduction, primary and secondary metabolite biosynthesis, phytohormone biosynthesis, photosynthesis and stress responses, to name a few. Based on the properties of *SlGRAS24* and the phenotypes of *SlGRAS24*‐OE transgenic plants, we focused on genes involved in pollen development, hormonal biosynthesis/signalling and genes encoding transcription factors (Table 4). A total of 11 genes were selected for supplementary qRT‐PCR analysis, including 3 pollen‐related genes, 5 hormone‐related genes and 3 transcription factor genes which were stamen development regulators (Fig. 8). For all the genes tested, qPCR analysis results validated the transcriptomic data.

**Table 4:** Nonexhaustive list of genes differently regulated (*P* < 0.05) between wild‐type and *SlGRAS2*4‐OE tomato anthesis flowers. Genes indicated with asterisks were validated by qPCR

	ITAG 2.40 Tomato	*Arabidopsis* orthologue	Functional annotation	Log2 fold (OE L10/WT)	Log2 fold (OE L15/WT)
Pollen development‐related genes	Solyc01g008680.2		S2 self‐incompatibility locus‐linked pollen 3.2 protein	−1.39	−1.31
	Solyc01g008740.1	AT5G12180	CDPK17, involved in pollen tube growth	−1.43	−1.2
	Solyc01g059910.2*	AT3G51550	Mediates male–female gametophyte interactions during pollen tube reception	−2.12	−1.47
	Solyc01g067370.2*		Pollen‐specific lysine‐rich protein SBgLR	−1.26	−1.26
	Solyc02g076860.2	AT4G18596	Pollen allergen Phl p 11	−1.2	−1.37
	Solyc05g026360.2	AT5G56750	Pollen‐specific protein SF21	−1.31	−1.16
	Solyc06g008240.2	AT4G04900	RIC10, involved in pollen tube growth	−1.44	−1.11
	Solyc06g008650.2	AT5G42232	Pollen allergen ole e 6	−1.21	−1.43
	Solyc09g065450.2		Pollen allergen ole e 6	−1.45	−1.2
	Solyc10g081700.1*	AT3G11690	Pollen preferential protein	−1.26	−1.31
	Solyc12g014240.1	AT1G29140	Pollen ole e 1 allergen and extensin	−1.16	−1.23
	Solyc12g014580.1		Pollen allergen Ole e 6	−1.14	−1.29
	Solyc10g081460.1	AT5G36940	CAT3, involved in amino acid transport	−1.1	−1.06
	Solyc09g010090.2	AT3G52600	Cell wall invertase 2, involved in sucrose catabolic process	−1.33	−1.23
	Solyc01g107830.2	AT3G53160.1	UDP‐glycosyltransferase superfamily protein	−2.66	−3.4
Hormone signalling‐related genes	Solyc05g025920.2	AT5G20810	SAUR‐like auxin‐responsive protein	−1.55	−1.59
	Solyc06g063060.2	AT1G54070	Auxin‐repressed protein‐like protein	−1.58	−1.28
	Solyc09g056360.2*	AT3G25290	Auxin‐responsive family protein	−1.05	−1.21
	Solyc10g011660.2	AT2G46370	Auxin‐responsive GH3 family protein	−1.29	−1.18
	Solyc12g009280.1*	AT5G20810	SAUR‐like auxin‐responsive protein	−1.22	−1.37
	Solyc10g009640.1*	AT2G46370	Jasmonic acid‐amido synthetase JAR1	−1.52	−1.33
	Solyc03g044740.2	AT3G50440	Methyl jasmonate esterase	1.11	2.08
	Solyc12g009220.1*	AT1G19180	Jasmonate ZIM‐domain protein 1 (JAZ1)	1.68	2.25
	Solyc00g095860.1	AT4G08040	ACS11, involved in ethylene biosynthetic process	1.92	2.2
	Solyc10g076450.1*	AT3G51770	Ethylene‐overproduction protein 1	−1.32	−1.31
	Solyc10g076320.1	AT5G01700	PP2C, ABA response	−1.4	−1.28
	Solyc07g005680.2	AT4G30610	BRI1 SUPPRESSOR 1 (BRS1), involved in brassinosteroid signalling via BRI1	−1.28	−1.45
	Solyc12g008900.1	AT1G75450	Cytokinin oxidase/dehydrogenase 2	1.04	0.98
Transcription factors	Solyc01g079260.2	AT2G47260	WRKY transcription factor 4	1.15	1.28
	Solyc01g087990.2*	AT5G13790	MADS‐box transcription factor 3	−1.17	−1.02
	Solyc01g094320.2	AT2G45800	GATA type zinc finger transcription factor family protein	−1.19	−1.28
	Solyc01g100460.2	AT1G75390	Basic‐leucine zipper (bZIP) transcription factor	−1.4	−1.34
	Solyc02g073580.1	AT4G18650	Basic‐leucine zipper (bZIP) transcription factor	2.29	2.1
	Solyc03g116890.2	AT1G80840	WRKY transcription factor 2	2.82	3.17
	Solyc04g009440.2	AT5G63790	NAC transcription factor	1.61	1.84
	Solyc04g056360.2*	AT4G26440	WRKY transcription factor 78	−1.45	−1.58
	Solyc05g051830.2	AT1G22130	MADS‐box transcription factor 1	−1.44	−1.56
	Solyc06g034030.2	AT5G56840	MYB‐like transcription factor family protein	−1.55	−1.24
	Solyc06g068570.2	AT1G16060	AP2‐like ethylene‐responsive transcription factor	1.39	1.15
	Solyc08g006470.2	AT2G24500	C2H2 zinc finger protein FZF	1.86	1.64
	Solyc08g008280.2	AT5G24110	WRKY transcription factor 30	1.77	1.83
	Solyc09g015770.2	AT3G56400	WRKY transcription factor 6	1.49	1.17
	Solyc09g014990.2	AT5G56270	WRKY‐like transcription factor	2.4	2.67
	Solyc11g020950.1	AT1G58110	Basic‐leucine zipper (bZIP) transcription factor	−1.41	−1.05
	Solyc11g044740.1	AT1G10200	GATA type zinc finger transcription factor family protein	−1.21	−1.265
	Solyc11g045310.1	AT2G03060	MADS‐box transcription factor	−1.29	−1.23
	Solyc12g044610.1*	AT3G16350	MYB transcription factor	−1.33	−1.24

## Discussion

The miR171‐GRAS regulatory network participates in various physiological processes, including shoot meristem maintenance, axillary bud formation, flowering time, chlorophyll biosynthesis and trichome distribution. Tomato plants underexpressing *SlGRAS24* did not differ from WT, while overexpression of *Sly‐miR171* did cause plants to grow taller and flower earlier (Fig. 2A), providing another clue about the functional redundancy among GRAS family members or *miR171* target genes. Indeed, it has been shown the existence of functional redundancy among GRAS proteins in *Arabidopsis*. GRAS protein SCL3 mutant line *scl3‐1* does not show any phenotype when compared with WT, but it confers important role in promoting gibberellin signalling by antagonizing master growth repressor DELLA protein (Zhang *et al*., 2011). Loss function of only one of the three *miR171* target genes in *Arabidopsis* does not have visible effects on plant growth, while transgenic plants overexpressing MIR171c (35S‐109mmpro*MIR171c*) and *scl6‐II scl6‐III scl6‐IV* triple mutant plants exhibit a similar reduced shoot branching phenotype (Wang *et al*., 2010). On the other hand, overexpression of *SlGRAS24* caused alteration of many important agronomical traits such as plant height, flowering time, leaf architecture, inflorescence architecture, lateral branch number, root length, fruit set and development (Table 2), making it a good target gene for generating new elite crop varieties with optimal flowering time and plant architectures, thus to meet the increasing demand for food, feed and biofuel production. Interestingly, we found that the plant height of *SlGRAS24*‐OE lines was severely suppressed at the early development stage, but the suppression gradually disappeared due to the growth of later branches. Overexpression of *SlGRAS24* led to apical dominance inhibition and this inhibition accompanied the whole life cycle of the OE lines. At the mean time, because of apical dominance inhibition, the lateral branches showed vigorous growth, in number and length. As we know, it has long been a target of breeding selection to affect tiller and panicle/ear branching complexity because it significantly affects crop yield (Springer, 2010).

The relationship between GA and GRAS proteins has been extensively studied (Sun *et al*., 2012), but some GRAS proteins have also been found to be involved in auxin signalling. For instance, an auxin‐induced GRAS protein AtSCL15 plays crucial role during seed maturation programme (Gao *et al*., 2015). The SCR/SHR complex comprised of two GRAS members has been proved to take part in root development by modulating both GA and auxin signalling (Heo *et al*., 2011; Rovere *et al*., 2015; Zhang *et al*., 2011). Interactions between GA and auxin are established by intricate crosstalk and self‐regulatory mechanisms involving the expression of auxin transport and GA metabolism genes. However, many important aspects of this relationship remain undiscovered. Plant hormones are implicated in the growth and development of shoot apical meristem (SAM) and root apical meristem (RAM) (Benková and Hejátko, 2009; Perilli *et al*., 2012; Shani *et al*., 2006). Important roles have been attributed to GA/auxin signalling in SAM and RAM (Weiss and Ori, 2007). It has been documented that *HAM* genes are required for the maintenance of both SAM and RAM (Engstrom *et al*., 2011), suggesting that *HAM* genes might exert functions in SAM and RAM by modulating GA and auxin signalling. In *Arabidopsis*, although it is found that *Atham1, 2, 3* exhibits root apex auxin maxima that are comparable to WT in spatial expression and intensity (Engstrom *et al*., 2011), it did not link *AtHAM* gene action directly to auxin signalling. Here, we demonstrate that tomato SlGRAS24 is a key transcription factor for coordinated regulation of GA and auxin signalling pathways, which may provide clues to the mechanisms underlying the significantly increased height of *Atham1, 2, 3* mutant plants and their shorter primary roots (Engstrom *et al*., 2011; Wang *et al*., 2010). *Atham1, 2, 3* mutant plants also have altered lateral branch formation (Schulze *et al*., 2010; Wang *et al*., 2010), a process which is also regulated by auxin and GA (Martínez‐Bello *et al*., 2015; Müller and Leyser, 2011; Ni *et al*., 2015). Recently, it was found that overexpression of a sunflower (*Helianthus annuus* L.) *GRAS‐*like gene altered the GA content and axillary meristem outgrowth of transgenic *Arabidopsis* plants (Fambrini *et al*., 2015). Tomato plants overexpressing *SlGRAS24* were dwarf with short primary roots, fewer lateral roots and more lateral branches (Fig. 2 and Table 2). Thus, it is plausible that *HAM* genes are regulators of endogenous GA/auxin balance in SAM, RAM and axillary meristems to control meristem maintenance and organ production.

Although *cis*‐acting elements including AuxRE and GARE were found in the promoter region of *SlGRAS24* gene and *SlGRAS24* transcripts were up‐regulated under exogenous GA_3_ and IAA treatment (Fig. 5), we observed that most GA/auxin‐related genes were significantly down‐regulated in *SlGRAS24*‐OE tomato seedlings. This was despite these seedlings containing more endogenous IAA than WT (Fig. 6B) and raises the possibility that there is a negative feedback loop between *SlGRAS24* expression and auxin metabolism. The reduced auxin responsiveness of *SlGRAS24*‐OE seedlings under IAA or NAA treatment (Fig. 7) implies that *SlGRAS24* might function in the downstream signalling response rather than in the upstream biosynthesis. There are more than ten kinds of active GAs in plant (GA9, GA4, GA34, GA7, GA51, GA19, GA20, GA1, GA8, GA5, GA3, GA29, etc.). Although the GA3 was elevated in OE seedlings (Fig. 6B), we do believe that the total GA content is reduced since most GA synthetic genes were down‐regulated (Fig. 6A) and the observation of dwarfism phenotype and lower seed germination rate. Noticeably, the expression of *SlDELLA*, which acts as repressor of GA signalling, was down‐regulated (Fig. 6). In contrast to the slender and earlier flowering time phenotype exhibited by *pro* mutant (a point mutation in the *DELLA* gene) tomato plants (Carrera *et al*., 2012 **)**, dwarfism and late flowering time phenotypes were observed in *SlGRAS24*‐OE plants, indicating the inhibition of DELLA‐dependent GA responses. Moreover, application of GA_3_ to *SlGRAS24*‐OE plants only partially rescued the dwarf phenotype and the germination rate to the WT level (Fig. 7), suggesting that these typically GA‐related phenotypes are not merely due to the alteration of the GA signalling.

As transcription factors, GRAS proteins have been shown to participate in various biological processes in dozens of higher plant species. However, less is known about their roles during reproductive developmental stages. In lily (*Lilium longiflorum*), a GRAS protein named LiSCL was found to be involved in microsporogenesis (Morohashi *et al*., 2003). In *Arabidopsis*,* Atham1, 2, 3* mutants occasionally produced flowers with three or five petals during the very early stages of flowering (Wang *et al*., 2010), which indicated the potential active roles of miR171‐targeted *GRAS* genes in flower organ formation. Here, we found that *SlGRAS24* played pivotal roles in late stamen development. *SlGRAS24* transcripts accumulated most in flowers during anthesis, predominantly in stamens (Fig. 1A and B). Overexpression of *SlGRAS24* in tomato impaired pollen sac and pollen development and as a consequence the fruit set ratio was lower than in WT (Fig. 3 and Table 2). Pollen development in flowering plants is a highly programmed process requiring many genes. In the nonexhaustive list of genes differentially regulated between *SlGRAS24*‐OE and WT flowers (Table 4), 15 are related to pollen development, 13 are associated with hormones and 19 are transcription factors. Most phytohormones play crucial roles in the regulation of pollen development either directly (Song *et al*., 2013) or indirectly (Dobritzsch *et al*., 2015; Ji *et al*., 2011). Our results demonstrated altered expression of genes related to all hormones except GA (Table 4). Young tomato flower buds have a high content in metabolites of GA pathways, which decrease progressively during ovary development. Active GA levels are very low in anthesis‐stage flowers during pollination, after which the total GA content within the ovary increases again (Fos *et al*., 2000). As we only compared the transcriptome of flowers undergoing anthesis, it is understandable why GA‐related gene expression did not differ between WT and *SlGRAS24*‐OE lines. Many transcription factors are involved in regulating pollen development in a dynamic regulatory network. In our work, the transcription factors differentially regulated by *SlGRAS24* are mainly from MYB, WRKY, MADS, zinc finger and bZIP gene families. In *Arabidopsis*, a number of MYB proteins have been documented as important regulators of pollen development (Cheng *et al*., 2009; Higginson *et al*., 2003; Mandaokar and Browse, 2009; Mandaokar *et al*., 2006; Yang *et al*., 2007). Two pollen‐specific transcription factors, WRKY34 and its close homolog WRKY2, are required for male gametogenesis (Guan *et al*., 2014). A subset of pollen‐specific MIKC*MADS box proteins (AGL30/65/66/94/104) are expressed preferentially during pollen maturation and double mutant combinations reveal the important roles these genes play in pollen germination and pollen fitness (Verelst *et al*., 2007). Interestingly, we found decreased expression of *Solyc04g056360.2* and *Solyc11g045310.1*, which are the homologous gene of *Arabidopsis WRKY34* (AT4G26440) and *AGL30* (AT2G03060, a member of AtMIKC*MADS complexes), respectively. It has been proved that *WRKY34* is one of the direct target genes of AtMIKC*MADS complexes in pollen. During pollen maturation, *WRKY34* is suppressed by several MIKC*MADS box transcription factors (Verelst *et al*., 2007). Thus, we speculate that *SlGRAS24* might participate in the MIKC*MADS‐WRKY34 regulatory network in tomato pollen development.

Upon flower fertilization, fruit and seed development occurs concomitantly, orchestrated by various phytohormones (McAtee *et al*., 2013). In *SlGRAS24*‐OE transgenic plants, fruits were smaller with fewer seeds (Fig. 3A). It is believed that pollen quantity and/or quality are closely associated with fruit and seed set (Aizen and Harder, 2007; Burd, 1994). In normal conditions, the developing seed continually sends signals to the surrounding tissue to expand and there is usually a positive correlation between seed number and fruit size (Nitsch, 1970). Therefore, we assumed that overexpression of *SlGRAS24* led to impaired pollen sac development and pollen viability, which resulted in less efficient pollination/fertilization and hence smaller fruits with fewer seeds. Both cell division and cell elongation programmes were significantly suppressed in the smaller transgenic fruits (Fig. 4), as would be predicted by the ‘seed control’ hypothesis that the seeds communicate through hormones to the surrounding tissue(s) to promote fruit growth firstly through cell division and then cell expansion (Ozga *et al*., 2002).

## Experimental procedures

### Plant materials and growth conditions

Tomato plants (*Solanum lycopersicum* cv. Micro‐Tom) were grown on soil in controlled glass house conditions with 14‐h light: 10‐h dark cycles, 25 °C day: 20 °C night temperature, 60% relative humidity and weekly irrigation with plant nutrient solution. For gene expression analysis, roots, stems and leaves were collected from 1‐month‐old plants, and flowers were harvested at the bud and anthesis stages and fruits at the immature, breaker and red stages. Receptacles, sepals, petals, stamens and ovaries were harvested from flowers at the anthesis stage. For each tissue/organ type, samples were collected from at least six healthy plants, mixed and then frozen in liquid nitrogen immediately. Sampling was done three independent times.

### Plasmid construction and generation of transgenic plants

Four DNA fragments, the *SlGRAS24* promoter, the precursor of *miR171*, the full‐length *SlGRAS24* coding sequence and a partial *SlGRAS24* coding sequence were amplified from tomato genomic DNA or cDNA. Primer sequences used for amplification are listed in Table S1. The *SlGRAS24* promoter sequence was fused with GUS in an expression vector. The *miR171* precursor and *SlGRAS24* full‐length coding sequence were cloned into the modified binary vector pLP100 in the sense orientation, while the partial *SlGRAS24* coding sequence was cloned in the antisense orientation, all under the CaMV 35S promoter. Transgenic plants were generated by *Agrobacterium tumefaciens*‐mediated transformation according to Huang *et al*. (2016). For each construct, more than 6 independent lines with consistent phenotypes were obtained. Homozygous lines from T2 or later generations were used for experiments.

### Subcellular localization and transactivation activity assay of SlGRAS24

The *SlGRAS24* open reading frame without the stop codon was amplified and cloned into the pGreen0029 vector. The recombinant plasmid containing the *SlGRAS24*‐*GFP* fusion gene and the control plasmid with *GFP* alone were transformed into tobacco (*Nicotiana tabacum* L.) protoplasts according to Ren *et al*. (2011). For transactivation assays, the coding region of *SlGRAS24* was amplified and ligated into the yeast expression vector pGBKT7 (Clontech, Japan) to produce pBD‐*SlGRAS24*. According to the manufacturer's instructions, pBD‐*SlGRAS24*, pGBKT7 (plasmid for negative control) and pGBKT7‐53+pGADT7‐T (plasmid combination for positive control) were transformed separately into the yeast strain AH109. Transformants were selected on SD/‐Trp or SD/‐Ade/‐His/‐Trp medium (Clontech, USA). The transactivation activity of each protein was evaluated by comparing growth on permissive and selective medium and the activity of X‐α‐Gal (5‐bromo‐4‐chloro‐3‐indoxyl‐α‐d‐galactopyranoside).

### Histochemical and histological analysis

GUS activity was assayed by submerging plant samples in 0.5 mg/mL X‐Gluc solution (0.1 m sodium phosphate buffer pH 7.0, 10 mm EDTA, 0.1% Triton X‐100, 0.5 mm potassium ferrocyanide, 0.5 mm potassium ferricyanide), infiltrating them under vacuum and incubating them at 37 °C. Samples were destained in 70% ethanol.

Histological preparations were performed according to Gabe (1968). Specific tissues/organs were embedded in FAA solution (50% (v/v) ethanol, 5% (v/v) acetic acid and 3.7% (v/v) paraformaldehyde). Samples were then placed under vacuum for 10 min, incubated at room temperature for 24 h, then dehydrated in an ethanol gradient and embedded in paraffin (Paraplast Plus, Sigma). Observations were carried out under a light microscope (OLYMPUS BX‐URA2, Japan).

### Hormone treatments for gene expression analysis

Two‐month‐old WT tomato plants were sprayed with 100 μm GA_3_ or 100 μm IAA. Leaves were harvested 0, 1, 3, 6, 12 and 24 h after spraying, frozen in liquid nitrogen and stored at −80 °C until RNA extraction. Meanwhile, 15‐d‐old pro*SlGRAS24*‐*GUS* transgenic seedlings were soaked in liquid MS/2 medium containing 20 μm GA_3_ or 20 μm IAA for 3 h, and then the whole seedlings were used for GUS staining analysis or directly frozen in liquid nitrogen and stored at −80 °C. Similarly, 10‐d‐old WT and *SlGRAS24*‐OE transgenic seedlings were soaked in liquid MS/2 medium containing 20 μm GA_3_ or 20 μm IAA for 3 h, and whole seedlings were frozen in liquid nitrogen and stored at −80 °C. Seedlings soaked in liquid MS/2 medium without hormone were used as controls. All treatments were performed three independent times.

### Hormone treatments for analysis of plant development

Surface‐sterilized seeds of WT and each T2 transgenic tomato line were germinated in the dark. After the emergence of the radicle, seeds were transferred onto MS/2 medium containing 10^−6^ m IAA and/or 5 × 10^−5^ m GA_3_. The seedlings were grown in a controlled growth chamber for 2 weeks with a 16‐h light: 8‐h dark photoperiod and 25 °C day: 20 °C night temperature. For auxin dose–response experiments, cotyledon explants from 10‐d‐old WT and *SlGRAS24*‐OE seedlings were incubated on MS/2 medium containing the indicated auxin (α‐naphthalene acetic acid, NAA) concentrations in the same growth chamber conditions for 10 d.

For seed germination assays, surface‐sterilized seeds from T2 transgenic lines and WT were germinated on MS/2 medium containing 10 μm GA_3_ or 5 μm paclobutrazol (PAC), a GA biosynthesis inhibitor, then placed in the same growth chamber conditions described above. Seeds germinated on MS/2 medium were used as controls. Germination rates based on radicle tip emergence were scored daily for 7 d after sowing. GA_3_ was also applied by spraying the aerial part of 10‐d‐old plants grown in the glasshouse with 20 μm GA_3_ solution containing 0.1% Tween‐80 every other day for 3 weeks. Control plants were sprayed with the equivalent solvent solution.

All experiments were repeated three times with about 30 seeds or 10 plants for each genotype.

### Extraction and quantification of endogenous IAA and GA_3_

Pure IAA and GA3 were purchased from Sigma Chemical Co. (St Louis, MO). Isotopically labelled internal standards [^2^H_5_]IAA and [^2^H_2_]GA_3_ were purchased from ICON Isotopes (Summit, NJ). Plant hormones were extracted and quantified as previously described (Pan *et al*., 2008) with some modifications. Approximately 0.2 g of 10‐d‐old WT or *SlGRAS24*‐OE seedlings was frozen in liquid nitrogen and ground into powder. Two millilitres of isopropanol–HCl buffer solution (2:0.002, v/v) was added to the powder and shaken for 30 min at 4 °C. Dichloromethane (4 mL) was added, shaken for 30 min and then centrifuged at 13 000 ***g*** for 5 min. After centrifugation, the lower organic phase was transferred to a 10‐mL tube and evaporated in a constant stream of nitrogen. Each sample was kept in the dark and resolubilized in 150 μL methanol containing 0.1% formic acid, and then the solution was filtered using a 0.45 μm microfilter for HPLC‐MS/MS analysis. Samples were injected into a reversed‐phase column (C18 ZORBAX 300SB 3 μm, 4.6 × 150 mm, Agilent, CA) using a binary solvent system composed of methanol (solvent A) and water with 0.1% formic acid (solvent B) as the mobile phase. The column thermostat was set at 30 °C. Separations were performed using a solvent gradient which started at 20% methanol for 2 min, increased linearly to 80% over 14 min and maintained for 5 min, and returned to 20% methanol for 0.1 min, when it was allowed to equilibrate for 5 min. A hybrid triple quadrupole/linear ion trap mass spectrometer (SCIEX 6500 QTrap, Applied Biosystems, Foster City, CA) was used with nebulizer gas pressure set at 75 psi, drying gas pressure at 65 psi, curtain gas pressure at 15 psi, source voltage at 4.5 kV and source temperature at 500 °C.

### Pollen viability assay

Pollen activity was evaluated by soaking pollen grains in 0.1% 2, 3, 5‐triphenyl‐2 h‐tetrazolium chloride (TTC) solution. Viable pollen stains red because the NADH/NADPH produced reduces TTC to 1,3,5‐triphenylformazan (TPF), which is red. Stained pollen grains were observed under the microscope.

### Digital gene expression profiling

Total RNA was extracted from anthesis‐stage flowers of *SlGRAS24*‐OE plants (lines 10 and 15) and WT controls using RNeasy^®^ Plant Mini Kit (Qiagen) following the manufacturer's protocol for RNA‐Seq. RNA quantity and quality were assayed in the Agilent 2100 Bioanalyzer (Agilent Technologies). Two independent RNA samples from transgenic or WT plants were sent to Illumina Cluster Station and Illumina HiSeq^™^ 2000 System (BGI Inc.) for RNA library construction and deep sequencing. RSeQC‐2.3.2 program (http://code.google.com/p/rseqc/) was used to assess the quality of RNA‐Seq data. Clean tags were obtained after quality filtering of sequences and mapped to the annotated genome sequence of *S. lycopersicum* in the Tomato Sol Genomic Network database (http://solgenomics.net/), and transcript abundance was also normalized by the fragments per kilobase of exon per million mapped reads (FRKM) method using Cuffdiff software (http://cufflinks.cbcb.umd.edu/) to identify differentially expressed genes (DEGs). The expression level of a gene from RNA‐Seq was normalized by the tags per million method. The criteria for defining differentially expressed genes were a false discovery rate (FDR) <0.05 with a *P* value <0.05. The raw transcriptome reads reported here have been deposited in the NCBI Short Read Archive under accession no. SRA473616.

### Real‐time quantitative PCR

One microgram of total RNA (RNeasy^®^ Plant Mini Kit, Qiagen) was used to synthesize first‐strand cDNA (RevertAid^™^ First Strand cDNA Synthesis Kit, Fermentas). Transcript levels were determined by absolute qPCR according to the methodology described in Huang *et al*. (2015) using specific primers. Primer sequences used for qRT‐PCR are listed in Table S2. Amounts of mRNA in samples were quantified using three biological replicates.

### Statistical analysis

All data in this report were obtained from at least three independent experiments with three technical replicates each. For data analysed with Student's *t*‐test, the differences between treatments were considered as significant when *P* < 0.05.

## Supporting information


**Figure S1.** Increased lateral branches (red arrow) and abnormal flower buds emergence (white arrow) in *SlGRAS24*‐overexpressing (*SlGRAS24*‐OE) transgenic tomato plants.
**Figure S2.** Representative fruits of WT and *SlGRAS24*‐OE plants at 3 dpa, 7 dpa, and 15 dpa. dpa, days postanthesis.

**Table S1.** Primer sequences used for amplification.
**Table S2.** Primer sequences used for qRT‐PCR analyses.

**File S1.** DEGs between WT and OE L10 anthesis flowers.

**File S2.** DEGs between WT and OE L15 anthesis flowers.
