# Supplementary material for: Overexpression of a tomato miR171 target gene SlGRAS24 impacts multiple agronomical traits via regulating gibberellin and auxin homeostasis
Source: Plant Biotechnol J. 2016 Nov 4;15(4):472–88. doi: 10.1111/pbi.12646 (PMC5362688; doi:10.1111/pbi.12646)

Figure S1. Increased lateral branches (red arrow) and abnormal flower buds emergence (white arrow) in SlGRAS24-overexpressing (SlGRAS24-OE) transgenic tomato plants

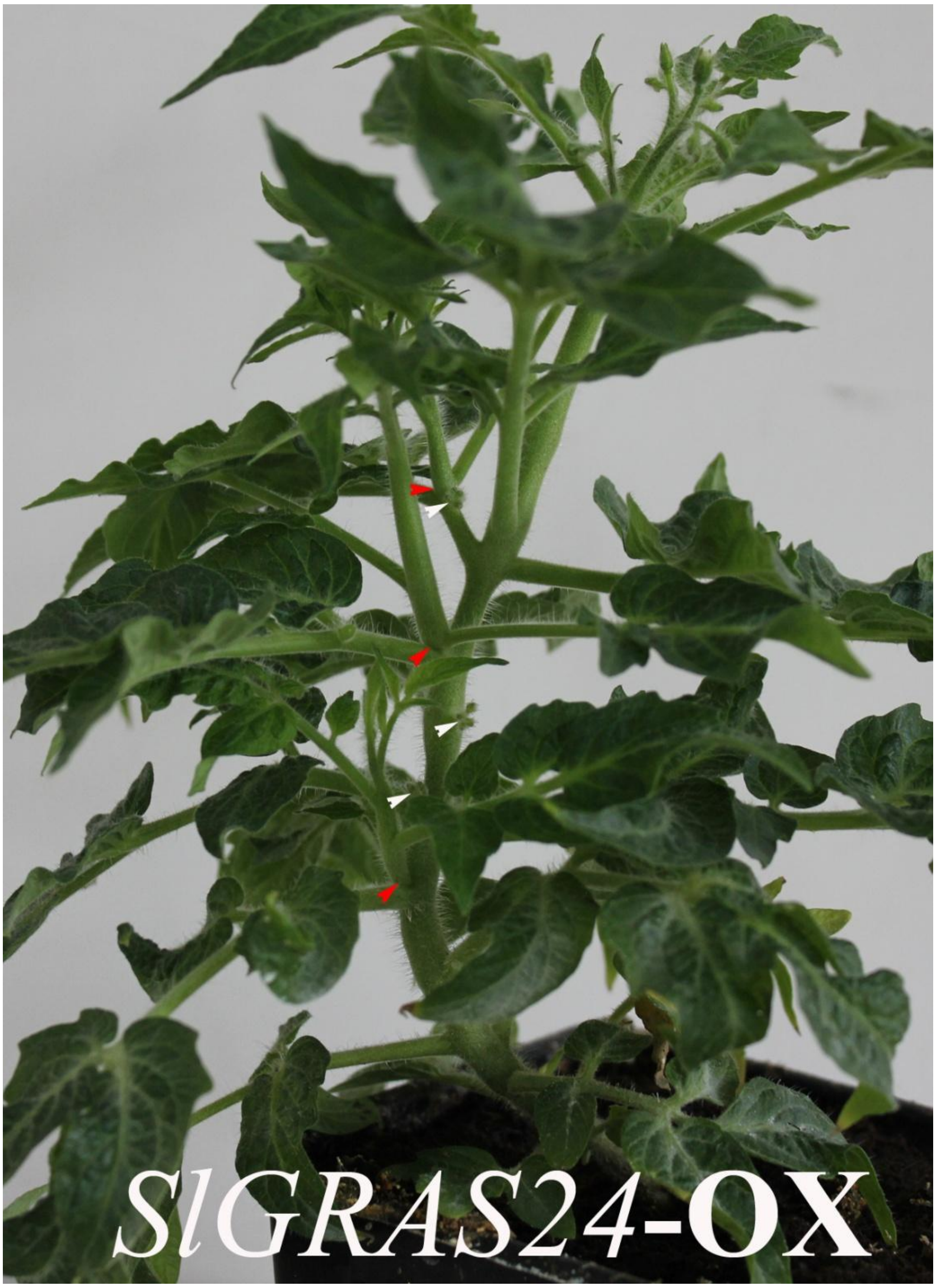

Figure S2. Representative fruits of WT and *SlGRAS24*-OE plants at 3 dpa, 7 dpa, and 15 dpa. dpa, days post anthesis.

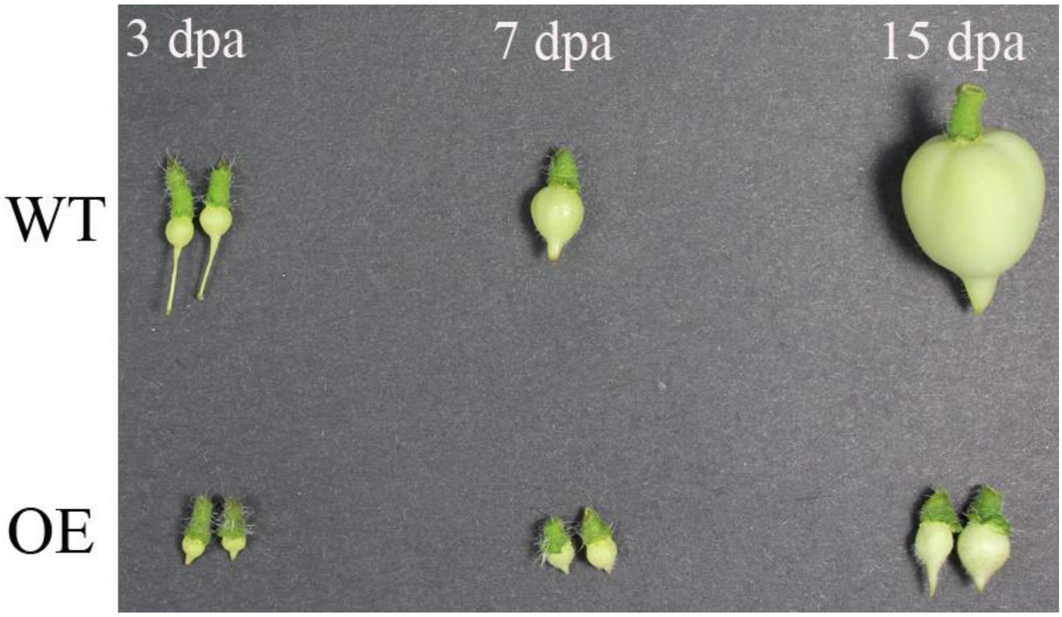

Supplement: Supplementary file 1 — Figure S1. Increased lateral branches (red arrow) and abnormal flower buds emergence (white arrow) in SlGRAS24‐overexpressing (SlGRAS24‐OE) transgenic tomato plants. Figure S2. Representative fruits of WT and SlGRAS24‐OE plants at 3 dpa, 7 dpa, and 15 dpa. dpa, days postanthesis. [file PBI-15-472-s004.pdf]
