# Supplementary material for: Overexpression of a tomato miR171 target gene SlGRAS24 impacts multiple agronomical traits via regulating gibberellin and auxin homeostasis
Source: Plant Biotechnol J. 2016 Nov 4;15(4):472–88. doi: 10.1111/pbi.12646 (PMC5362688; doi:10.1111/pbi.12646)
Supplement: Supplementary file 2 — Table S1. Primer sequences used for amplification. Table S2. Primer sequences used for qRT‐PCR analyses. [file PBI-15-472-s001.pdf]

Tabel S1 Primer sequences used for amplification.

Primer name	PCR primers	
	Forward (5'-3')	Reverse (5'-3')
SlGRAS24	TGAATGAGGTGGGTGTCCTTAG	TGCCAGCAGAGAACAAGTGA
SlGRAS24-as	TCAAGCCCTTCAGTCCTACTCA	CCGAAACAATCTGCCTATGC
SlGRAS24-GFP	TATGAATGAGGTGGGTGTCC	ACAGGAGTCCTCTTGACTACACA
pre*Sly-miR171*	GTTGGTGCGGTTCAATGAGA	TAACAACAACATCGGAGGCG
pro*SlGRAS24*	TGAAAATGCAGGCTTGGTGCT	CCTTCTGATTTCTGCCATGGA

Tabel S2 Primer sequences used for qRT-PCR analyses.

Primer name	PCR primers	
	Forward (5'-3')	Reverse (5'-3')
q*SlGRAS24*	TGGTCCAGACAGGGAATCCG	TTCAAGTTGTTGGTGTTGGCA
q*SlFT*	GCAAGCACAGGAGTAACCTTTGG	CGGGAGCATAGATGATTTCTCG
q*SlCO1*	TGCCTCGCTTTGTGCCTCT	CCATCATCCTCGGTGCCTT
q*GUS*	GTGATGTCAGCGTTGAACTGCG	GGTTGCCAGAGGTGCGGATT
q*SlGA20ox1*	CTCATTTCTAATGCTCATCGT	TGCAGATGATTCTTTCTTAGCG
q*SlGA20ox2*	TTTCCATATTCTACCCTACAAG	TCATCGCATTACAATACTCTT
q*SlGA20ox4*	GATGATAAATGGCACTCTATTC	TGACTTCCTTGTTCTTCTACAG
q*SlGA2ox1*	GGCATGTAAGATATTAGAATTGA	TTAATCCGTAGTAGAGAATCAGA
q*SlGA2ox2*	ATTAAGATCCAATAACACTTCG	TCTTGATTTCACACTATTTGC
q*SlGA2ox4*	ATGGAAGGAAAAGACAGTTTA	CTTTTCTCAAATAGGACCAAC
q*SlGA3ox1*	GGCATTAGTAGTTAATATAGGTGA	AAATAAGCTACAGAAAGTCGATA
q*SlGA3ox2*	GATCATAAATTTGTCATGGATAC	TGTTTCCATATGGTTAAGTAATC
q*SlDELLA*	TGATGCGACTATACTTGATATAAG	GGGTTAATCTGTTTAATAGAGTTC
q*SlIAA2*	TAACAATGATGAACCACCAC	TAACAATGATGAACCACCAC
q*IAA4*	AACAAGAGGGCTTTGCCTGAG	GTGTCTTGGCAACAGGTGGA
q*IAA7*	ACTCAACCTCCATCATAATGATAATATTCC	ACCCCACCACTTGAGCCTTA
q*IAA9*	CCCCTTGCACCCTTCCA	AGCGTCTGAAAATCCTCGTTTG
q*SlPIN1*	GCTGCAGGCTGGTCTAGATT	AACAATGGCAACAAAGCACA
q*SlPIN3*	TTCAAAATCAATTTAGCGTGTCA	CTCAAAATCCCTCTTGTTTCG
q*SlPIN5*	ACATTGAGCTGGCATTTTGG	TCCACTACCAGCCTTTGACA
q*SlPIN6*	AGATGGCAGCAATAGGGATG	GCGAAGACAAATGGAACGAT
q*SlARF5*	ATTAGTTCTGAGTTGTGGC	GGTATCTGTGAAGTTGCTG
q*SlARF6*	GGTTCAACGGGTTCTCAAC	TTCAGGGAAGTGGATGCTC
q*SlARF7*	CCAAGTTATCCTAATCTTCCTTCC	GTAAAGCCTCCTGGTCATATTTG
q*SlARF8*	CTGCTCAAACCCAAATGCTGTC	GGTAAGTGTGTTGGTGAGCCTG
q*SlCDKB2.1*	TGGTGTATTGCACAGGGATCTGAAA	ACTTCTTAATGGGCAGAGTATAAGC
q*SlCycB2.1*	GTGGAAACAAGAAGCAACCGAA	TGCATATTTCCTCGTGATTGGTC
q*SlCycD3.1*	TTTAGATTCCCAGCTCCAAAATCCTA	TTCATTTCCGACATCTAAACTAGACC
q*SlXTH1*	TGGGCTAGTCACCATATTAAGTTC	CTCTGCATTATTCGATGACAGGT
q*Solyc09g056360.2*	CGTTGCCGTTATTCTTGTCTTTG	GCAGCATCGTATGTCCAATGAA
q*Solyc12g009280.1*	ATAAGCAAGAGGCTACGGAGC	TATCCTTTCGGGACGCCA
q*Solyc10g009640.1*	GCAGGCAAGCACTTAGTTGACG	GCATCCAGGAACGATTTGTCC
q*Solyc12g009220.1*	CATCGTCGTCCGTTGAAACA	TGGTCATTTGTGCCTTCTCTGG
q*Solyc10g076450.1*	CAGGCTGATTGCTGGATGC	GCTCTGTGGCTGTTCAACCG
q*Solyc01g059910.2*	GGTTCAGTGCTGTGGAATCTTGA	TGTCCTCCGCCGACTCCTTA
q*Solyc01g067370.2*	TTTCTGTTGTTGCTGTTGTGGAG	CTTCTTCCTTCTCTTCCGCTTTC
q*Solyc10g081700.1*	AGTCCGATTGCGATGGTCG	CACTTCGTTGAGATGGGCTCC
q*Solyc01g087990.2*	GCGTGCTGGCTTGTTGAA	GCTGCTCGTTGTCCTCTGACTT
q*Solyc04g056360.2*	TGTGAAAGGCAACCCGAATC	CCGTAGCACCATTTGTCTTGTTT
q*Solyc12g044610.1*	CAGCAGAACCATCACGGCA	TCGGAGCAGTTTCACCCTTG
q*SlUBI*	GCCGACTACAACATCCAGAAGG	TGCAACACAGCGAGCTTAACC
q*miR171*(RT)	CTCAACTGGTGTCGTGGAGTCCGGCAATTCAGTTGAGGATATTGG	
q*miR171*	ACACTCCAGCTGGGTGATTGAGCCG	AACTGGTGTCGTGGAGTCCGG
U6	TCTAACAGTGTAGTTTGTCCCTTCG	TTGTGCGTGTCATCCTTGC
